# Ayahuasca-assisted meaning reconstruction therapy for grief: a non-randomized clinical trial protocol

**DOI:** 10.3389/fpsyt.2024.1484736

**Published:** 2025-01-07

**Authors:** Pablo Sabucedo, Oscar Andión, Robert A. Neimeyer, Oscar Soto-Angona, Julia Javkin, Josep Maria Haro, Magi Farré, Débora González

**Affiliations:** ^1^ Faculty of Health and Life Sciences, University of Liverpool, Liverpool, United Kingdom; ^2^ Sociedad Española de Medicina Psicodélica (SEMPsi), Barcelona, Spain; ^3^ Research Sherpas, Girona, Spain; ^4^ Portland Institute for Loss and Transition, Portland, OR, United States; ^5^ Parc Sanitari Sant Joan de Déu, Barcelona, Spain; ^6^ Institut de Recerca Sant Joan de Déu (IRSJD), Barcelona, Spain; ^7^ Department of Psychiatry and Forensic Medicine, Universitat Autonoma de Barcelona, Cerdanyola del Vallés, Spain; ^8^ Kiyumi Collective, Hoosfdorp, Netherlands; ^9^ Heart & Brain Training, Nijmegen, Netherlands; ^10^ PHI Association, Barcelona, Spain; ^11^ Center for Biomedical Research in Mental Health Network, (CIBERSAM), Madrid, Spain; ^12^ Department of Clinical Pharmacology, Hospital Universitari Germans Trias I Pujol-IGTP, Badalona, Spain; ^13^ Departament of Pharmacology, Therapeutics and Toxicology, Universitat Autonoma de Barcelona, Cerdanyola del Vallés, Spain; ^14^ Faculty of Health Sciences, Isabel I University, Burgos, Spain

**Keywords:** psychedelic-assisted therapy, ayahuasca, psychedelics, meaning reconstruction, psychotherapy, protocol, bereavement, prolonged grief disorder

## Abstract

**Background:**

Psychotherapy for Prolonged Grief Disorder (PGD), a condition characterized by an intense and persistent grief response, has received increased attention over the past decades. Evidence-based approaches to prevent PGD are currently scarce, and not always effective. This paper introduces a protocol for a clinical trial exploring the effectiveness of a Meaning Reconstruction psychotherapy approach (MR) assisted with ayahuasca, a traditional indigenous medicine.

**Method:**

The outlined protocol is a three-arm, non-randomized controlled trial focused on reducing normal and pathological grief symptoms, comparing the effectiveness of Ayahuasca-assisted MR therapy (A-MR), MR therapy alone (MR) and No Treatment (NT). At least 69 people who lost a first-degree relative during the prior year, and with a Texas Revised Inventory of Grief score up 39 (TRIG ≥ 40), will participate in the trial. Participants will be allocated to an A-MR (n ≥ 23), MR (n ≥ 23) or NT (n ≥ 23) group. Those from the A-MR and MR therapy groups will undergo a therapeutic process involving 9 sessions of online psychotherapy. In addition, the A-MR condition involves 2 group sessions of ayahuasca. The primary outcomes will be normal and pathological grief severity as measured by the TRIG and Traumatic Grief Inventory Self-Report (TGI-SR), administered at baseline, post-treatment, and 3-month follow up. Measures of quality of life, post-traumatic growth, meaning-made, psychological flexibility, and self-belief consistency will be also included. In addition, subjective effects of ayahuasca and acceptance-avoidance promoting effects will be assessed following ayahuasca administration. Finally, we will analyze the potential mediating effect of meaning-made, psychological flexibility and self-belief consistency in grief symptoms (as measured by the TRIG and TGI).

**Discussion:**

This trial is the first to empirically examinate the potential of psychedelic-assisted psychotherapy for grief, as well as the potential processes of change that may account for it.

**Clinical trial registration:**

https://clinicaltrials.gov, identifier NCT06150859.

## 1 Introduction

Grieving the death of a loved one is a nearly universal experience, and one which may be experienced multiple times over the course of a lifetime. Recent studies have shown that, for each death, approximately nine relatives are directly affected (1). The loss of a first-degree family member significantly increases the risk of mortality, disease, and suicide for the mourner (2–6). Although grief is a natural process, and serves a key existential purpose (7), this psychological process can go awry. Over the last century, different theories have explored how grief itself can become complicated or pathological.

Prolonged Grief Disorder (PGD) has been recently incorporated as a diagnosis into the ICD (ICD-11; 8) and the DSM (DSM-5-TR; 9). PGD is characterized by (1) separation distress (i.e. a persistent grief response involving intense longing and preoccupation), (2) intense emotional reactivity (e.g. anger, denial, blame, emotional numbness, meaninglessness, avoidance), and (3) significant functional impairment (10, 11) according to the mourner’s sociocultural context. This reaction should persist to a marked degree for a minimum of 6 months, following the ICD-11 (8), or 12, months following the DSM-5-TR (9).

The inclusion of PGD in the psychiatric diagnostic system is not without controversy. Besides the difficulties of distinguishing normal from prolonged grief, and the arbitrariness involved in categorizing continuous variables such as distress or time (10), it has been argued that the requirement for these diagnoses may lead to people experiencing acute grief not receiving appropriate healthcare (12). On the other hand, people in Western countries are rapidly losing the cultural knowledge, tradition, and rites to cope with the death of a loved one (13). In this social context, imposing a 6-to-12-month wait for receiving evidence-based psychological support can leave bereaved people abandoned in the most vulnerable state, especially for those at moderate or high health risk.

Despite the WHO’s emphasis on the importance of preventive strategies for addressing the global burden of mental health issues (14), and on loneliness as a significant public health issue (15), evidence-based approaches to prevent PGD are still scarce. Moreover, meta-analyses have shown that preventive therapies for PGD are not currently effective (16, 17). Innovation is therefore necessary to provide effective psychotherapeutic and community support to people in the acute period of grief (18).

### 1.1 Psychedelic-assisted grief therapy

Psychedelic-assisted psychotherapy (PAP), a therapeutic approach augmenting talking therapy with psychedelic substances, has been recently classified as a potential breakthrough treatment for psychiatric illness (19). PAP is demonstrating promising outcomes in treating a wide range of mental health issues, ranging from depression to post-traumatic stress disorder, suggesting this efficacy may be transdiagnostic (20, 21). Controlled studies to date have been primarily focused on MDMA-assisted therapy for PTSD (22) and psilocybin-assisted therapy for treatment-resistant depression (23), but it has been proposed that psychedelics could also serve as a prophylactic or preventive intervention with the potential to improve quality of life, well-being, creativity, and personal and spiritual growth (24, 25).

Ayahuasca, a traditional indigenous medicine, has been investigated as a potential treatment for treatment-resistant depression (26). Several studies have evidenced that individuals who regularly use ayahuasca in a communal setting exhibit a higher level of general well-being, increased physical activity, and balanced diet than the general population (27–29). While ayahuasca may not cause physical or psychological dependence, and serious adverse effects are uncommon in healthy individuals (30), some users have reported experiencing psychological distress during its acute effects (31). In a global survey involving 10,836 participants, 2.3% reported requiring medical support following ayahuasca use (32). Additionally, there have been a few documented instances where its consumption has been associated with the emergence of psychotic episodes (33). Nevertheless, most adverse effects can be mitigated by conducting a thorough assessment of participants’ health status prior to administration, ensuring the composition and purity of the ayahuasca preparation, administering an appropriate dosage, and avoiding potential drug interactions (30) (see Section Potential Adverse Effects Management).

Despite the promising outcomes reported in the treatment of several mental health issues, no controlled studies have been conducted testing the efficacy of PAP for PGD, nor have any evaluated PAP’s potential in preventing prolonged grief when administered in the early course of bereavement. Previous observational research, however, has suggested that the ceremonial use of ayahuasca has therapeutic value in reducing the severity of grief (34–36). We therefore propose that ayahuasca assisted with an evidence-informed therapeutic approach, the meaning reconstruction model (37, 38), could show greater reduction in grief severity than psychotherapy alone.

The rationale behind this hypothesis is grounded in a wide range of neurobiological and psychological evidence. Ayahuasca is an Amazonian concoction, usually prepared from the *Banisteriopsis caapi* vine and the *Psychotria viridis* shrub, which has been utilized for healing and ritual purposes since pre-Columbian times (39). Regarding the neurobiological evidence, *in vitro*, animal and human studies have demonstrated how harmine, tetrahydroharmine, harmaline (all three present in *B. caapi*) and dimethyltryptamine (present in *P. viridis*) stimulate adult neurogenesis and neuroplasticity, similarly to other serotoninergic psychedelic substances (40–43). A window for neuroplastic changes would appear to open a few hours after administration, lasting from a few days to a month (44). This period has been related to improvement in grief, depressive mood, stress, mindfulness, acceptance, emotion regulation, social behavior, wellbeing, and quality of life (45). Moreover, elevated peripheral Brain-Derived Neurotropic Factor (BDNF) levels have been observed in healthy volunteers and clinically depressed patients two days following the ayahuasca administration, correlating with clinical improvement in those who were diagnosed with major depression (40). Recent studies have shown that psychedelics reopen a critical period to induce metaplasticity, which dynamically regulates the potential for synaptic plasticity (46, 47). This critical period is context-dependent and has been linked to the ability to induce social reward learning, psychological flexibility, cognitive reappraisal and new patterns of behavior (46, 48, 49). In sum, these studies highlight the importance of the post-treatment integration period to maximize the therapeutic effect of psychedelics.

Looking beyond the neurobiological evidence, several studies indicate that the therapeutic effect is experience-dependent (50, 51). Current research points toward specific traits of the psychedelic experience as relevant mediators of the therapeutic outcomes of PAP, such as acceptance-related experiences, the presence of mystical experiences, or the reappraisal of negative emotion (52–55). The altered state of consciousness induced by psychedelics is characterized by high sensitivity to the immediate and previous context, which can help actualize of the person’s belief system with bottom-up information (56). The ayahuasca experience, therefore, could bring new information to the foreground to enrich the therapeutic process, providing an opportunity to transform maladaptive narratives about oneself, other people (including the deceased) or the world (56, 57). The preparatory psychotherapeutic work, moreover, could bring to light potential blockages or resistances, allowing for an appropriate preparation of the patient’s mindset and intention before the administration of ayahuasca. Lastly, psychotherapy following ayahuasca administration (including, but not limited to, psychedelic integration) could likewise help to promote new behavior, in a way that is congruent with reappraised cognitive schemas derived from the assimilation and accommodation of the experience during this critical period.

We posit, in sum, that ayahuasca experiences, when taking place within the framework of an appropriate and effective psychotherapeutic intervention, could enrich the clinical treatment of grief. This would allow for the integration of new information evoked from the psychedelic experience, and for this to result in more adaptive behavior and coping. We also hypothesize this would favor grief adaptation through fostering psychological processes such as meaning reconstruction, experiential acceptance, or cognitive reappraisal during a period of heightened meta-plasticity.

## 2 Study protocol

### 2.1 Study design and hypotheses

This three-arm, non-randomized controlled trial investigates the efficacy of Ayahuasca-assisted Meaning Reconstruction therapy (A-MR) in decreasing normal and pathological grief symptoms in people who have lost a first-degree relative within the prior 12 months. Participants are sequentially allocated to the following treatment conditions: (1) a control group receiving Meaning Reconstruction therapy (MR), (2) a control group receiving No Treatment (NT), and (3) an experimental group receiving Ayahuasca-assisted Meaning Reconstruction therapy (A-MR).

The objective of this trial is two-fold. First, our aim is to compare the efficacy of A-MR with that of MR and NT by assessing differences in (a) the severity of normal grief symptoms, (b) pathological grief symptoms, (c) quality of life, and (d) posttraumatic growth. Moreover, we are also interested in analyzing how each condition may influence the proposed processes of change: (e) meaning-made (f) psychological flexibility, and (g) self-belief consistency. Additionally, we will explore the participants’ (h) subjective effects of ayahuasca and the (i) acceptance-avoidance promoting effects.

The following hypothesis will be tested:

Hypothesis 1. The experimental group receiving A-MR will show greater improvement in normal and pathological grief symptoms, quality of life, and post-traumatic growth compared to both control groups (MR and NT), at post-treatment and 3 months follow-up. Moreover, MR group will show greater improvement than the NT group, expecting a medium clinical effect size.

Hypothesis 1.1. The experimental group receiving A-MR will show greater improvement in psychological flexibility, meaning-made, and self-believed consistency compared to both control groups (MR and NT), at post-treatment and 3 months follow-up. Furthermore, MR will also show greater improvement than the NT group, expecting a medium clinical effect size.

Hypothesis 2. Reductions in the severity of normal and pathological grief symptoms will be principally related to changes in meaning- made, but also to changes in psychological flexibility and self-belief consistency. Moreover, a stronger relationship between changes in the severity of grief symptoms and meaning-made is expected in the A-MR group.

### 2.2 Participants

Participants eligible for this study will be English and Spanish-speaking adults experiencing grief symptoms due to the death of a loved one. Inclusion criteria involve: (1) people aged 18-65; (2) having experienced the loss of a first-degree relative; (3) within the past 12 months; and (4) with a Texas Revised Inventory of Grief score over 39 (TRIG ≥ 40). Exclusion criteria involve: (1) medically and mentally significant health conditions; (2) history of psychotic disorder (Axis I- DSM-IV-TR); (3) pregnant or breastfeeding women; (4) hypertension (SBP > 140 mmHg, DBP > 90 mmHg, HR >100 bpm); (5) substance use disorder, excluding nicotine; (6) alcohol consumption exceeding 40g/day; (7) concurrent psychological or pharmacological treatment for grief. Those who are not eligible will be referred to other mental health professionals specialized in bereavement.

### 2.3 Procedure

**Figure 1:**
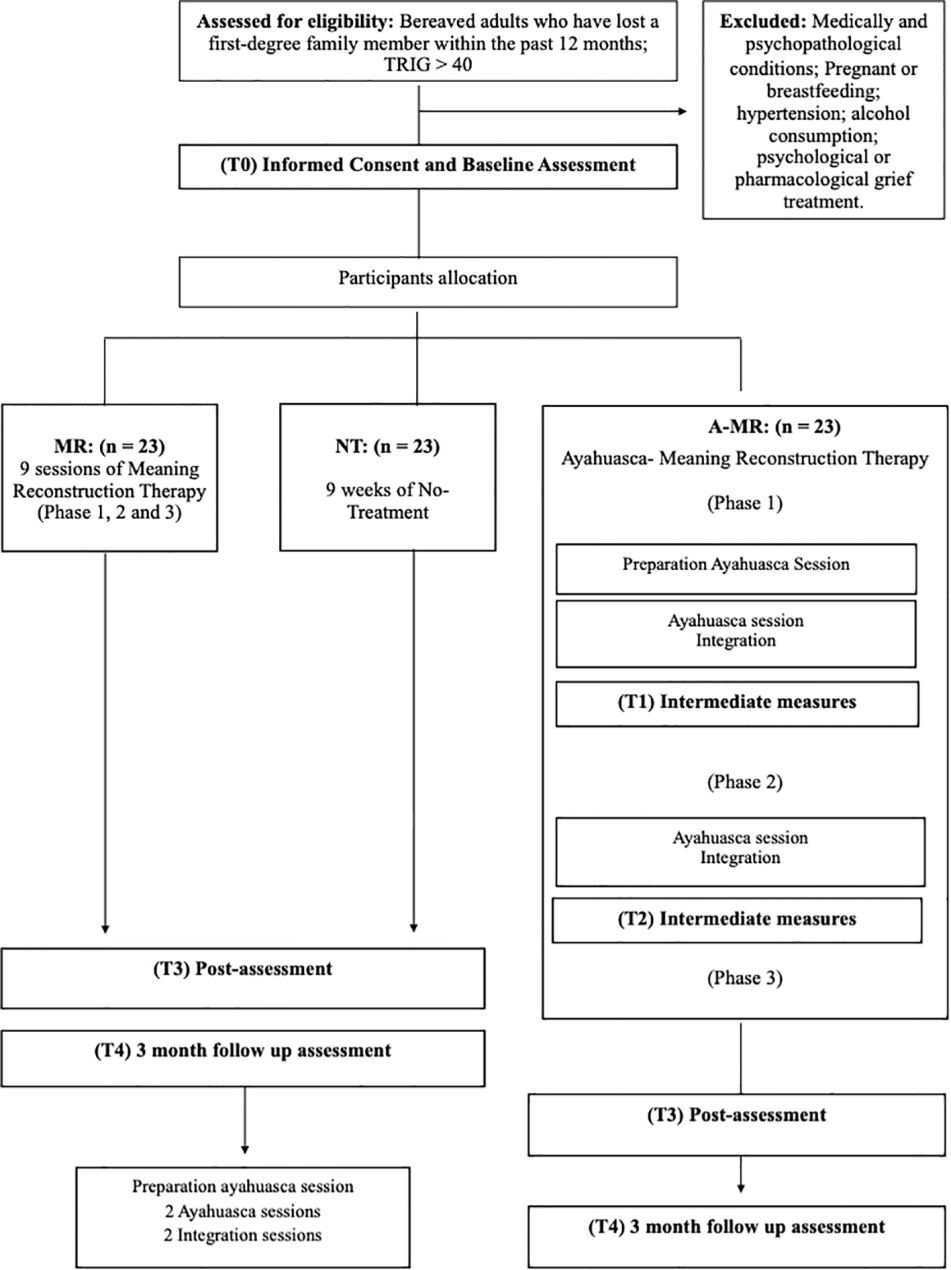
Flow-chart of the recruitment procedure.

Participants will be recruited through the newsletter, webpage, and social media accounts of the Beckley Foundation, Beckley Med, MIND Foundation and the International Center for Ethnobotanical Education, Research and Service (ICEERS), all of them European institutions dedicated to psychedelic research and education. Upon receiving the request to participate in the trial, the participant will be prompted to complete a short online questionnaire to screen for inclusion criteria. Those who meet the inclusion criteria will attend a video-call interview with the principal investigator (DG) to learn more about what their participation would entail and to screen whether any exclusion criteria are met. They will also be informed about the need to verify their blood pressure range with a general practitioner to prevent potential ineligibility, given that blood pressure measurement is taken on-site before each ayahuasca session. Then, those who are eligible will provide informed consent. Assessments will be conducted online at baseline (T0), during treatment (T1 and T2), at post-intervention (T3), and at 3-month follow-up (T4) (see **Figure 1**).

Participants in the A-MR and MR group will undergo a 9-session online psychotherapy process, starting within 2 weeks after the baseline assessment is completed. Only those in the A-MR group will participate in up to two onsite ayahuasca sessions throughout the therapeutic process, preceded by an online preparation session, and followed by a 50-minute MR online integration session during the week after each ayahuasca experience. For ethical reasons, participants in either control group (MR or NT) have the option to participate in two ayahuasca sessions over a single weekend, once follow-up assessment is completed, if their grief symptom severity are still significant (TRIG ≥ 40), proceeded by a preparation session and followed by two integration sessions to process the experience. **Table 1** shows the measures used at each of the time points.

**Table 1:** Enrolment, intervention, and assessment schedule (SPIRIT).

	STUDY PERIOD									
	Enrolment	Baseline	Allocation	Treatment					Post-treatment	Follow-up
TIMEPOINT		(T0)		Phase 1	Ayahuasca (T1)	Phase 2	Ayahuasca (T2)	Phase 3	(T3)	(T4)
ENROLMENT										
Eligibility screen	x									
Informed consent	x									
Allocation			x							
INTERVENTION										
MR				x		x		x		
NT										
A-MR				x	x	x	x	x		
ASSESSMENT										
TRIG	x	x							x	x
TGI-SR		x							x	x
PSGI-SF		x							x	x
WHO-qol-BREF		x							x	x
AAQ-II		x							x	x
ISLES-SF		x							x	x
SCS		x							x	x
5D-ASC					x		x			
APEQ					x		x			
SA									x	

MR, Meaning Reconstruction Therapy; NT, No treatment; A-MR, Ayahuasca-assisted Meaning Reconstruction Therapy; TRIG, Texas- Revised Inventory of Grief; TGI-SR, Traumatic Grief Inventory self-report; PSGI-SF, Post-traumatic Growth Inventory‐Short Form; WHO-qol-BREF, World Health Organization Quality of Life-Brief; AAQ-II, Acceptance and Action Questionnaire II; ISLES-SF, Integration of Stressful Life Experiences Scale Short-Form; SCS, Self-concept clarity Scale; 5D-ASC, Altered State of Consciousness Rating Scale; APEQ, Acceptance/Avoidance-Promoting Experiences Questionnaire Scale; SA, Level of Satisfaction and Adverse Effects

### 2.4 Sample size

Given that no comparative trials analyzing the therapeutic effect of PAP on reduction of grief symptoms has been published to date, sample size was calculated based on the results of González et al. (35) observational study. The observed Cohen’s d (0.84) for the study group between the baseline and the 15^th^-day-post-treatment assessment, and TRIG measurement correlation of 0.55, were used to estimate the sample size for this study. Therefore, sample size was calculated based on the assumption of a between groups differences at post-treatment of at least 9.4 points (TRIG score) in mean change between the A-MR and the NT groups. Sample size with power of 0.90 and an alpha of 0.05 was calculated using pwrss R package version 0.3.1 (58). To find at least the above effect size, a total sample of 69 participants is needed, but as drop-out rate of 20% were considered, 84 participants will be recruited.

### 2.5 Psychometric measures

#### 2.5.1 Primary outcomes

##### 2.5.1.1 Texas Revised Inventory of Grief (TRIG)

This self-report measure quantifies the severity of normal grief (59). In this trial, only one scale (the Present Feelings Scale, PFS) was used to assess the participant’s emotional state. This 13-item scale is scored on a 5-point Likert-type scale (from 1 = ”completely false” to 5 = ”completely true”) ranging from 13 to 65. Scores up to 39 (TRIG ≥ 40; above the 50th percentile), are defined as severe grief (60). The TRIG has been shown to have adequate psychometric properties, with the PFS having a Cronbach’s coefficient alpha value of.90 (61).

##### 2.5.1.2 Traumatic Grief Inventory Self-Report (TGI-SR)

A measure designed to assess markers of grief disorder as defined by the ICD-11 and the DSM- 5, using PGD criteria (62). This questionnaire focuses on a total of 18 symptoms experienced during the preceding month, assessed on a 5-point Likert-type scales (from 1 = “never” to 5 = “always”). A total score of ≥ 59 identifies those meeting criteria for PGD-caseness. Cronbach’s alphas of the TGI-SR in the patient sample were.95 (all 18 items),.95 (17 PCBD items), and.93 (11 PGD items).

#### 2.5.2 Secondary outcomes

##### 2.5.2.1 Posttraumatic Growth Inventory‐Short Form (PSGI-SF)

This questionnaire measures the degree of positive change experienced in the aftermath of a traumatic event (63, 64). The PSGI-SF is a 10-item self-report measure scored on a 6‐point Likert scale (from 1= “no change” to 6 = “a high degree of change”). The five subscales are: (1) relating to others, (2) new possibilities, (3) personal strength, (4) spiritual change, and (5) appreciation of life. Total scores ≥ 32 indicate probable personal growth (65). The internal consistency coefficient of the questionnaire is.86 -.89 (66).

##### 2.5.2.2 World Health Organization Quality of Life-BREF (WHOQOL -BREF)

This questionnaire is a shorter version of the original WHOQOL-100 (67). The measure was designed to assess quality of life in four different areas: physical health, psychological health, social relationships, and environment. For this study, only the first three scales were used. The 24-item questionnaire is scored on a 4–20 scale, with higher scores indicating a better quality of life. The scale has been shown to have adequate psychometric properties, with Cronbach’s coefficient alpha values ranging from.68 to.82 (68).

#### 2.5.3 Measures of potential mediators

##### 2.5.3.1 The Integration of Stressful Life Experiences Scale-Short Form (ISLES-SF)

This measure involves a self-assessment of “meaning made”, as the successful outcome of meaning-making after stressful life experiences (69, 70). ISLES-SF is a 6-item version of the original 16-item measure, and it relies on a 5-point Likert response scale (from 1 = “strongly disagree” to 5 = “strongly agree”). Scores above 14 on the ISLES-SF have been found to reflect clinically significant functional impairment in family, occupational and social contexts (71).The scale has a validated bi-factorial structure, permitting overall assessment of challenges to meaning-making, as well as scoring of two subscales: (1) *Footing in the World* (a measure of personal disorientation following loss) and (2) Comprehensibility (an index of ability to make sense of the experience; 72). The ISLES-SF has shown to have strong psychometric properties, with a Cronbach’s coefficient alpha value of.96 (73).

##### 2.5.3.2 Acceptance and Action Questionnaire (AAQ-II)

This is a unidimensional scale to assess psychological flexibility (74, 75). The measure relies on a 7-point Likert-type scale (from 1 =“ never true” to 7 = “always true”). The total score is calculated adding all individual scores, with lower scores corresponding to greater psychological flexibility. In terms of psychometric properties, the AAQ-II has good validity and reliability with the mean Cronbach’s alpha being.84

##### 2.5.3.3 Self-Concept Clarity Scale (SCS)

This 12-item scale measure the clarity and cohesiveness of the person’s self-concept (76). Self-concept clarity is measured on a 5 point-Likert scale (from 1= “strongly disagree” to 5 = “strongly agree”), with higher scores corresponding to greater self-concept clarity. The SCS has been shown to have adequate psychometric properties, with a Cronbach’s coefficient alpha value of .86.

#### 2.5.4 Measures of subjective effects

##### 2.5.4.1 The Altered State of Consciousness Rating Scale (5D-ASC)

This is a visual-analogue 94-item scale assessing five key dimensions of altered states of consciousness independently of their etiology (77). In this study, the 42-item version will be used (78), comprised of five different dimensions: (1) Oceanic Boundlessness, (2) Anxious Ego, (3) Visionary Restructuralization, (4) Auditory Alterations, and (5) Reduction of Vigilance (RV). The outcomes of the 5D-ASC data are percentage scores of maximum absolute scale values. This 42-item version has shown to have adequate psychometric properties, with Cronbach’s coefficient alpha of.83 for the 11-factorial structure (78).

##### 2.5.4.2 Acceptance/Avoidance-Promoting Experiences Questionnaire Scale (APEQ)

This psychometric instrument has been designed to measure experiences related to acceptance and avoidance, that occur during a psychedelic-induced altered state of consciousness. The questionnaire is self-reported and comprises 32 items, divided into two main scales: (1) the Acceptance-Related Experience (ACE) scale and the (2) Avoidance-Related Experience (AVE) scale, as well as two ancillary scales: Introspection and Interaction. The Acceptance-Related Experience (ACE) scale encompasses the subscales (1.1) Accepting Response, (1.2) Pro-Acceptance Insights, and (1.3) Relief. The Avoidance-Related Experience (AVE) scales comprise the subscales (2.1) Pro-Avoidance Insight, (2.2) Avoidant Response, and (2.3) Distress subscales. Cronbach’s coefficient alphas are.92 for the ACE scale,.89 for the AVE scale,.84 for the Introspection scale, and.75 for the Interaction scale.

#### 2.5.5 Level of Satisfaction and Adverse Effects Assessment (S.A)

In the post-assessment, participants will complete a survey comprising two satisfaction-related questions: (1) “To what extent are you satisfied with the therapeutic support provided during the psychotherapy sessions?”; (2) “To what extent are you satisfied with the ayahuasca sessions?”. Responses will be rated using a 5-point Likert-type scale (from 1 = “Very dissatisfied” to 5 = “Very satisfied”)

Adverse effects will be evaluated using the following questions: (3) “Have you experienced any negative effects from the psychotherapeutic sessions?”; (4) “Did you experience any negative effects during the ayahuasca session?”; (5) “Did you experience any negative effects in the week following the ayahuasca session?”. Responses will be categorized as: (1) No, (2) Uncertain, or (3) Yes.

Each of these questions will be followed by an open-ended prompt encouraging participants to provide further details about their responses.

### 2.6 Data analyses

All the studied variables will be descriptively analyzed by means (SDs), medians (ranges) or n (percentages). Between groups baseline differences will be tested using 2-tailed t-test, Man-Whitney test or chi-square/Fisher’s exacts test based on the type of variables compared.

Despite the trial being exploratory study, data will be analyzed following the intent-to-treat and/or completer strategies, based on the presence of drop-out. The first hypothesis (H1) will be tested using an independent two-way repeated measures analysis of variance (ANOVA) with 3 levels for each factor (Group: A-MR *vs* MR *vs* NT; Time: pre-treatment *vs* post-treatment *vs* follow-up) and/or Linear Mixed Models (LMM) for each primary and secondary outcome studied. The LMM will be used to analyze the possible effect of between groups differences at baseline measures as covariables, and to analyze the effect of the presence PGD diagnoses following the ICD-11 duration criterion at baseline. Bonferroni corrections will be applied to all analyses, and partial eta-square and Cohen’s *d* effect sizes will be calculated for interaction effects and for between- and within-group comparisons, respectively. Confidence intervals for Cohen’s *d* will be estimated at a 95% confidence level and 500 bootstrap samples.

The analysis of the second hypothesis (H2) will be based on the outcomes of testing H1, with reduction in grief severity (TRIG and TGI scores) as the dependent variable for mediation analyses. The mediation model will include all participants in the study and will only incorporate the potentially significant mediating variables (meaning-made, psychological flexibility, and self-belief consistency) identified in previous analyses. Power analyses for the mediation models will also be conducted based on a Monte Carlo simulation.

The statistical analyses will be conducted using R version 4.2.3 (79). The following R packages will be used for data exploration, description, and preprocessing if necessary: tidyverse (80), car (81), moments version 0.14.1 (82), and nortest version 1.0-4 (83). The repeated measures ANOVAs and/or Lineal Mixed Models will be conducted using rstatix package version 0.7.2 (84). Finally, the mediation model will be tested using the lavaan package version 0.6-12 (85) and statistical power for the mediational analysis will be calculated using WebPower package version 0.9.4 (86).

### 2.7 Intervention

#### 2.7.1 Meaning Reconstruction therapy

The psychotherapy protocol, part of both the A-MR and the MR group, is grounded in a meaning reconstruction approach to grief therapy (37, 38, 87). This model focuses on the crucial role of meaning-making in recovery and posttraumatic growth after the death of a significant person (69, 88–90). The intervention follows a tripartite approach that helps mourners overcome obstacles in making sense of the loss, reworking their attachment relationship to the deceased, and reconstructing their identities in light of the experience (37, 38). Open trials of the model with both unselected self-referred groups experiencing loss and transition (91) and mourners meeting criterial for PGD (92) have strongly supported the effectiveness of the model and, added to a growing literature, indicate that meaning-making prospectively predicts positive adaptation to bereavement (88, 92–95).

The 9-session A-MR and MR can be subdivided into the three domains defined in meaning reconstruction theory: the “event story” of the loss, the “back story” of the relationship with the deceased, and the “personal story” of self (37, 38) (**Table 2**):

1. The first phase is focused on the employment of non-directive narrative retelling to help the participant revisit the story of the loss, and the stressful or traumatic memories connected to it (37, 96, 97). This technique, when conducted in a safe and low-avoidance setting, can enable the bereaved to reconstruct a coherent narrative of the loss and its implications through introspection and deliberative processing and integration of its meaning. When necessary (e.g. when significant anxiety is present), relaxation and/or mindfulness techniques are introduced to manage activation and foster self-regulation.
2. The second phase is focused on promoting a secure attachment with the deceased (98), addressing unfinished business, or processing anger and guilt (37). Different techniques are employed to help reconstruct the internal representation of the deceased, such as introducing the deceased (99) and engaging in an imaginal conversation with them (100). When appropriate, and desired by the participant, a key aim is to establish and maintain a constructive continuing bond with the deceased (101).
3. The third and last phase prioritizes the reconstruction of the bereaved person’s sense of self and their future as a survivor (7, 102). The Bull’s-Eye Values Survey (BEVS), a contextual form of behavioral activation and values clarification, is employed to restore the participant’s life orientation without the deceased (103).

Participants will complete a 50-minute session on a weekly basis, conducted online through a secure videoconference platform in compliance with HIPAA guidelines. Every therapist in the trial is a licensed psychologist, and all of them have received specialized training in the delivery of the treatment protocol. Furthermore, they have access to clinical supervision provided by DG and PS upon request. All the therapists have prior experience in PAP and/or psychedelic integration and are affiliated with the Spanish Society of Psychedelic Medicine (SEMPSi).

#### 2.7.2 Ayahuasca-assisted Meaning Reconstruction therapy

The experimental group intervention (A-MR) adheres to the same online Meaning Reconstruction Therapy protocol, with the incorporation of up to two onsite ayahuasca sessions following phase 1 (session 3) and phase 2 (session 6) (see **Table 2**). The first ayahuasca session is preceded by a preparatory online group session (after session 1) and is followed by an online individual integration session during the week after the ayahuasca administration. This design is based on the contemporary structure of PAP, which typically involves a three-stage framework encompassing preparation, facilitation, and integration (104).

**Table 2:** Ayahuasca-assisted meaning reconstruction protocol (A-MR).

Phases	Psychological content	Session	Techniques
Opening	Fostering therapeutic alliance; Exploring potential difficulties; Psychoeducation: Dual model of grief coping	1	
On-line preparation of ayahuasca session*			
**Phase 1:** The “event story” of the loss	Processing traumatic or stressing memories, and finding meaning in loss and in life	2	Narrative retelling
		3	Narrative retelling
First ayahuasca session*			
Integration of ayahuasca experience*			
**Phase 2:** The “back story” of the relationship with the deceased	Restoring attachment security through reconstructing a bond with the deceased and/or processing unfinished business	4	Introducing the deceased
		5	Imaginal conversation with the deceased
		6	Internal representation of the deceased’s reconstruction
Second ayahuasca session*			
Integration of ayahuasca experience*			
**Phase 3:** The “personal story” of self	Reorganizing one’s identity and life orientation as a survivor	7	Bull’s-Eye Values Survey
		8	Bull’s-Eye Values Survey
Ending	Summary of the psychotherapeutic process	9	

*Excluded in the MR intervention protocol.

The objective of the preparation session is for participants to become familiarized with one another, address any uncertainties that might arise after reviewing the written information provided, and foster an appropriate expectation for the upcoming ayahuasca experience. Psychedelic integration following the ayahuasca session is conducted in alignment with the non-directive principles of restorative retelling (100, 105). Restorative retelling facilitates the assimilation of the phenomenology of the ayahuasca experience, while helping the person reconstruct their schemas and narratives about themselves, other people and the world, through a process of accommodation. A detailed outline of this method of psychedelic integration has been published elsewhere, in the form of a case study (106). Features of the psychedelic experiences that persist, or could gradually unfold, are further addressed during the remaining psychotherapeutic process.

##### 2.7.2.1 Ayahuasca session procedure

Each ayahuasca session is conducted onsite over a 24-hour period, starting on Saturday morning. At 11:00 am, each participant is asked to attend an initial group meeting to discuss how the day will be organized. Immediately after that meeting, blood pressure and body weight are assessed by a medical doctor to ensure that this inclusion criterion is met, and to calculate the appropriate ayahuasca dose for each participant.

At 13:00h, a second meeting takes place to practice mindfulness exercises in a group format, which are aimed at promoting a low-avoidance and present-focused mindset during the psychedelic experience. Before the start of the first ayahuasca session at 14:30h, each participant takes part in a one-hour group dynamic during which they introduce their loved one to the rest of group, by sharing photos of the deceased (99). During the second ayahuasca session, this activity is replaced by an art-therapy activity, the dual rose technique, which can aid in the reconstruction of meaningful memories of the deceased and the relationship with them (107). Both activities help anchor the psychological content related to the loss in the participants’ mindset, immediately before the ayahuasca administration takes place.

During the ayahuasca session in the afternoon, participants recline on a comfortable couch in a quiet, ample room, while listening to the same instrumental music through a loudspeaker. They are encouraged to focus their attention inward, and research staff are always present in the room to address participants’ needs. Psychedelic effects typically last 5 hours, approximately, and participants are encouraged to describe their experience in writing during the 2 hours immediately afterward. Those who prefer to paint or draw to assimilate and express their experiences, instead of writing them down, are provided with the materials to do so.

After this period of rest and reflection, a healthy vegetarian dinner is provided. Subsequently, participants sleep under medical supervision. The following morning, a light breakfast is served. Participants are then invited to optionally share their ayahuasca experiences in a group setting, expressing themselves as they feel comfortable. During this sharing, non-directive principles are followed to validate their psychedelic experiences. Before the session is over, they are provided with general information and advice regarding the sensitive state they may find themselves afterward, and how to manage it.

Ayahuasca sessions will be conducted in groups of up to eight participants, supported by a team of four facilitators. The primary responsibility of the facilitators is to ensure the physical and psychological safety and trust of participants throughout the session. They also provide non-directive support to help participants engage openly with their internal experiences as they emerge. The facilitator team includes the head of the Santo Daime Church (Barcelona), a psychiatrist (JJ), a nurse, and the principal investigator of the study (DG). All facilitators have extensive experience supporting individuals through altered states of consciousness. Their expertise spans religious settings, clinics treating treatment-resistant depression with ketamine-assisted psychotherapy, and several psychedelic research studies. They have also collaborated with retreat centers administering ayahuasca and psilocybin, in both traditional and non-traditional contexts.

##### 2.7.2.2 Potential adverse effects management

Ayahuasca has been demonstrated to be safe in naturalistic (35, 108) and clinical settings, across varying dosages and multiple administrations (26, 109–112). To enhance participant safety, individuals with a history of psychosis, medical complications, hypertension, or potential pharmacological interactions are excluded. While physically safe, the acute effects of ayahuasca are often challenging and not necessarily pleasant.

###### 2.7.2.2.1 Acute effects

Nausea and vomiting are common during ayahuasca sessions (32), so each participant will have a bucket, tissue, and water placed next to their mat to facilitate vomiting without needing to leave for the restroom. If a participant experiences significant distress, agitation, anxiety, fear, or paranoia, facilitators will provide reassurance and encourage acceptance and curiosity toward the challenging internal experiences. Grounding techniques, including deep breathing, mindful awareness (learned before the session), and progressive muscle relaxation, may also be suggested. Participants feeling overwhelmed by the group experience may be temporarily moved to a quiet space and remain under a direct facilitator’s supervision.

###### 2.7.2.2.2 Post-acute effects

The effects of ayahuasca are expected to last 5-6 hours, although participants may rest on their mats for up to 4 additional hours while listening to calming music under professional supervision. In cases of anxiety or distress, grounding techniques and emotional regulation strategies will be employed. If nausea or vomiting persists, antiemetic teas, such as ginger or chamomile, will be offered. Vital signs, including blood pressure, will be closely monitored in participants exhibiting adverse effects after the ayahuasca session.

###### 2.7.2.2.3 Rescue medication

After monitoring post-acute effects for 4 hours, the medical team will evaluate the need for rescue medication if clinically indicated. In cases of severe anxiety, 1 mg of lorazepam may be offered, and in cases of agitation or psychosis, 5 mg of olanzapine may be administered, with the option to repeat the dose after an hour if symptoms persist. In extreme cases of severe agitation, where there is a risk of self-harm or the participant hurting other people, intramuscular administration of 0.5 mg of clonazepam may be required. For cases of severe and persistent hypertension (SBP > 180 mm Hg, DBP > 120 mm Hg), participants will be closely monitored and lorazepam 1mg may be administered. If the hypertension persists after 30 minutes, 25 mg of captopril may be administered, with a second dose after 30 minutes if necessary. If systolic/diastolic blood pressure persists above 180/120mmHg, 10mg of amlodipine may be administered, with a subsequent referral to an emergency service to assess for target organ damage. Metoclopramide (10 mg) may be offered for severe, persistent nausea and paracetamol (500 mg) if the participant experiences severe headaches. Participants will remain under medical supervision for at least 12 hours after the ayahuasca experience: any residual symptoms will be addressed during subsequent psychotherapy sessions.

The excitement component of the Positive and Negative Syndrome Scale (PANSS-EC) (113) will be evaluated by the physician 24 hours after ingestion for all participants.

### 2.8 Pharmacological analyses and dosification

A 10-liter batch of ayahuasca (i.e, *Daime* in Portuguese) will be donated by CEFLURIS (IDARIS), a Brazilian-based religious organization associated with the Santo Daime church. Following a ritual tradition, ayahuasca is prepared by boiling the stems of *B. caapi* (which are rich in harmine, tetrahydroharmine, and harmaline) together with the leaves of *P. viridis* (which are rich in N,N-dimethyltryptamine) for several hours. Analyses are conducted by Energy Control (energycontrol-international.org) using Liquid Chromatography-Mass Spectrometry (LC-MS). The ayahuasca will be stored in a refrigerator at -40°C to minimize degradation of DMT and alkaloids as much as possible (114).

A medium dose of ayahuasca (0.75 mg DMT/kg) will be orally consumed in two different intakes (0.30 mg DMT/kg as an initial dose and 0.45 mg DMT/kg after 30 minutes). With this method, it is expected that the monoamine-oxidase-inhibiting properties of the β-carbolines in the first dose will block the metabolic degradation of DMT by MAO-A, enabling full access to systemic circulation once the second dose is administered (115).

## 3 Discussion

This clinical trial is the first to examine the potential of psychedelic-assisted grief therapy and, more specifically, of ayahuasca-assisted meaning reconstruction therapy for the reduction of the severity of grief symptoms. The study also investigates whether the intervention promotes quality of life and post-traumatic growth, while exploring a series of processes of change that may mediate this therapeutic effect. If this intervention proves to be both feasible and effective upon completion of this study, a future clinical trial will be designed with a focus on (a) prioritizing the mechanism(s) of change that will be identified through mediation analyses.

A key limitation of the trial is that the sequential allocation of participants may have a bias influence on the outcomes. Another limitation is a threat to external validity connected to the recruitment process: the self-referral process relies on the social media presence of psychedelic research and education institutions and associations, which may limit the generalizability of the clinical outcomes to the general population. Moreover, the fact that ayahuasca is a plant concoction complicates the determination of which alkaloids and active compounds influence the outcomes. Finally, accepting bereaved people who meet the ICD-11 sixth-month-since-the-loss criterion for PGD disorder (instead of the more conservative 12-month criterion) could blur the distinction between prevention and treatment. Nevertheless, we will also analyze PGD criteria following the ICD-11 as a potential moderator.

Given the lack of grief literacy in the general population, the widespread loneliness in Western society, and lack of mental health support during the early stages of bereavement, it is imperative to improve bereavement support for acute grief (13). The scarcity of evidence-based therapies for the prevention of PGD indicates the urgent need for new interventions to support bereaved people at moderate-to-high risk, according to the four-tier model (116). We anticipate that the outcomes of this trial will significantly contribute to the field of bereavement care, helping to reduce the time needed for the bereaved to receive evidence-based support, and promoting their adaptation. Moving beyond the context of grief and loss, this study will also contribute to advancing our understanding of PAP as way to prevent and reduce the global mental health burden (14).
