# Corrigendum: Ayahuasca-assisted meaning reconstruction therapy for grief: a non-randomized clinical trial protocol

**DOI:** 10.3389/fpsyt.2025.1590527

**Published:** 2025-04-01

**Authors:** Pablo Sabucedo, Oscar Andión, Robert A. Neimeyer, Oscar Soto-Angona, Julia Javkin, Josep Maria Haro, Magi Farré, Débora González

**Affiliations:** ^1^ Faculty of Health and Life Sciences, University of Liverpool, Liverpool, United Kingdom; ^2^ Sociedad Española de Medicina Psicodélica (SEMPsi), Barcelona, Spain; ^3^ Research Sherpas, Girona, Spain; ^4^ Portland Institute for Loss and Transition, Portland, OR, United States; ^5^ Parc Sanitari Sant Joan de Déu, Barcelona, Spain; ^6^ Institut de Recerca Sant Joan de Déu (IRSJD), Barcelona, Spain; ^7^ Department of Psychiatry and Forensic Medicine, Universitat Autonoma de Barcelona, Cerdanyola del Vallés, Spain; ^8^ Kiyumi Collective, Hoosfdorp, Netherlands; ^9^ Heart & Brain Training, Nijmegen, Netherlands; ^10^ PHI Association, Barcelona, Spain; ^11^ Center for Biomedical Research in Mental Health Network, (CIBERSAM), Madrid, Spain; ^12^ Department of Clinical Pharmacology, Hospital Universitari Germans Trias I Pujol-IGTP, Badalona, Spain; ^13^ Departament of Pharmacology, Therapeutics and Toxicology, Universitat Autonoma de Barcelona, Cerdanyola del Vallés, Spain; ^14^ Faculty of Health Sciences, Isabel I University, Burgos, Spain

**Keywords:** psychedelic-assisted therapy, ayahuasca, psychedelics, meaning reconstruction, psychotherapy, protocol, bereavement, prolonged grief disorder

In the published article there was an error in the **Funding** statement. The statement previously stated:

“The author(s) declare financial support was received for the research, authorship, and/or publication of this article. This study is sponsored by the Beckley Med Foundation. We wish to thank Christian af Jochnick, the Riverstyx Foundation, Christiana Musk and the Flourish Trust for their generous philanthropic support of this clinical trial”.

The correct funding statement appears in the one entitled “Manuscript Source.” This is the only change that needs to be made in the manuscript:

“The author(s) declare financial support was received for the research, authorship, and/or publication of this article. Financial support was received from Christian af Jochnick and the Multidisciplinary Association for Psychedelic Studies. The study is sponsored by the Beckley Med Foundation. We would like to express our gratitude to Christian af Jochnick, the Riverstyx Foundation, Christiana Musk, and the Flourish Trust for their generous philanthropic support of this clinical study.”

The authors apologize for this error and state that this does not change the scientific conclusions of the article in any way. The original article has been updated.

